# From Bouveret’s Syndrome to Gallstone Ileus: The Journey of a Migrating Stone!

**DOI:** 10.7759/cureus.2370

**Published:** 2018-03-26

**Authors:** Fady G. Haddad, Wissam Mansour, Joseph Mansour, Liliane Deeb

**Affiliations:** 1 Department of Gastroenterology and Hepatology, Staten Island University Hospital, New York; 2 Department of Pulmonary Critical Care, Staten Island University Hospital, Northwell Health; 3 Department of Radiology, Saint Louis University

**Keywords:** bouveret syndrome, gallstones, gall stone ileus

## Abstract

Bouveret’s syndrome, first described in 1896, is an unusual cause of gastric outlet obstruction secondary to large gallstone impaction in the proximal duodenum after migration through a cholecystoduodenal fistula. Stone migration has been previously described after endoscopic or surgical fragmentation. However, this is the first reported case, in our knowledge, where the stone migrated after oral contrast administration a few days after the onset of symptoms, causing a distal gallstone ileus.

## Introduction

Bouveret’s syndrome, first described in 1896, is an unusual cause of gastric outlet obstruction secondary to large gallstone impaction in the proximal duodenum after migration through a cholecystoduodenal fistula. Stone migration to the distal small intestine has been described after primary endoscopic or surgical fragmentation of the original impacted gallstone [1]. Our case report is the first, in our knowledge, to describe the migration of the duodenal stone after the oral administration of water-soluble hyperosmolar contrast agent, used while performing abdominal computed tomography (CT) imaging. This phenomenon occurred 48 hours after contrast intake and caused a distal jejunal gallstone ileus.

## Case presentation

**Figure 1:**
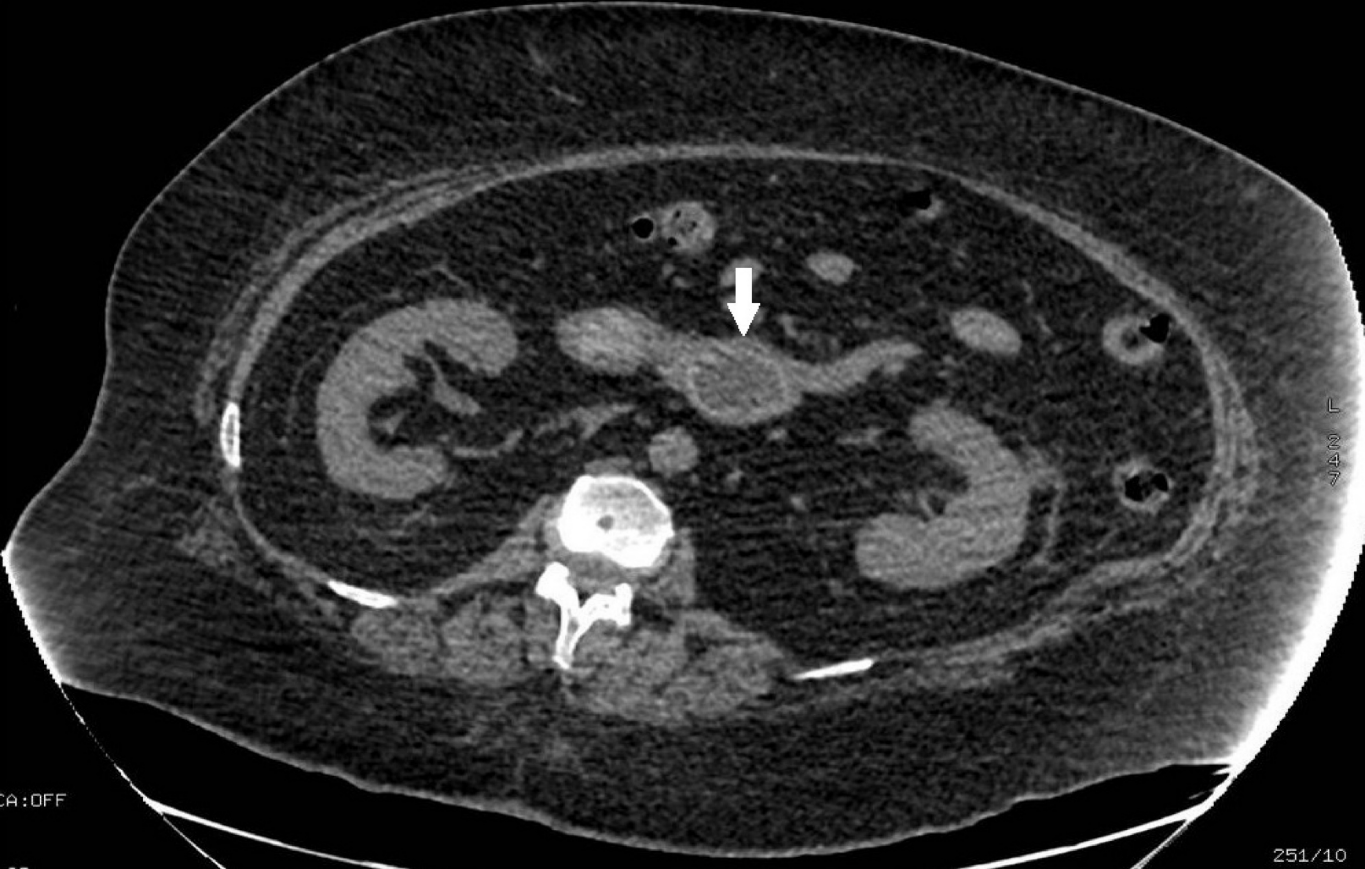
Abdominal CT scan axial cut showing the obstructing intraluminal stone in the duodenum (arrow) CT: computed tomography

A 61-year old female with diabetes mellitus and hypertension presented with a five-day history of epigastric pain and postprandial vomiting. A physical examination revealed a soft abdomen with mild epigastric tenderness. Labs were significant for an acute kidney injury. An abdominal CT scan following water-soluble hyperosmolar radiocontrast administration showed a 3.5 cm intraluminal calculus at the level of the third duodenal segment, diffuse pneumobilia, and an air-containing gallbladder, suggesting Bouveret’s syndrome (Figure 1).

**Figure 2:**
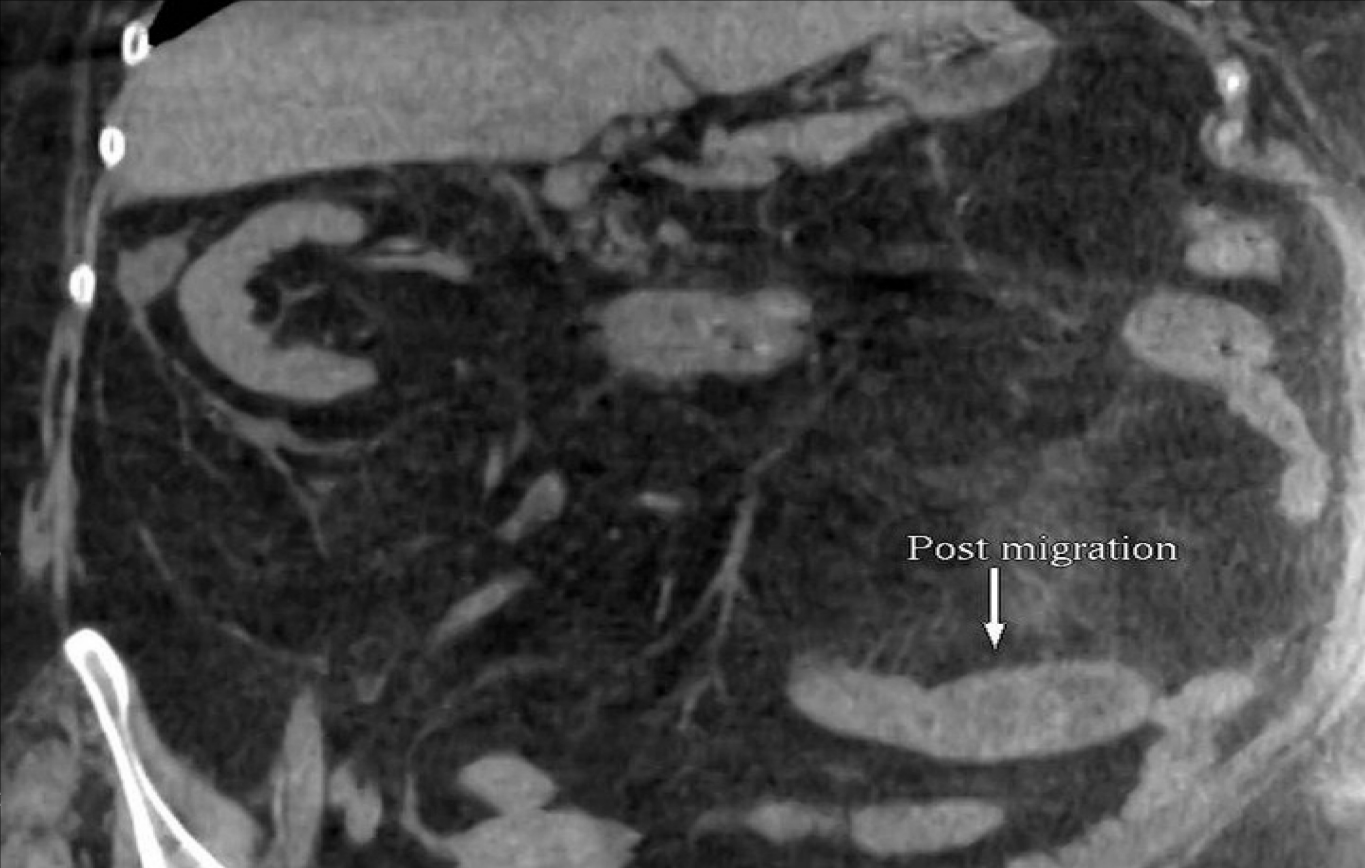
Abdominopelvic CT scan coronal cut showing the migration of the obstructing stone to the jejunum (arrow) CT: computed tomography

An initial improvement was noted later, followed by the recurrence of symptoms two days later. A repeat abdominal CT scan showed the migration of the duodenal stone and impaction at the distal jejunum with the dilation of the proximal loops, suggesting gallstone ileus (Figure 2).

**Figure 3:**
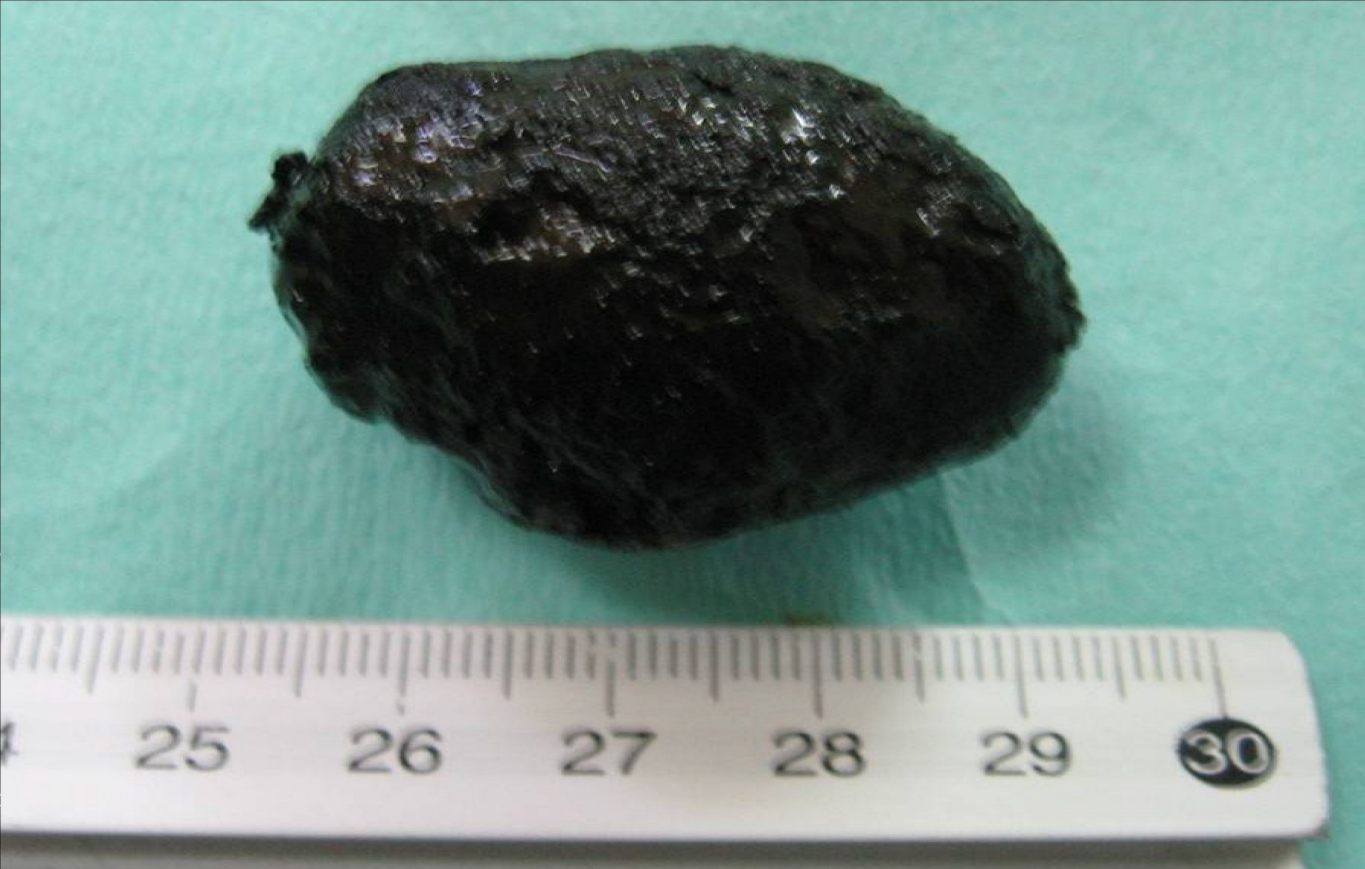
Extracted 4 cm gallstone following jejunotomy

Subsequent jejunotomy and the successful extraction of the stone (Figure 3) were performed, with no postoperative complications.

## Discussion

Although over 300 cases of Bouveret’s syndrome have been reported, it is unusual to see a migrating gallstone after seven days of obstruction. The rarity of stone migration and its timing after the ingestion of the water-soluble contrast raised the speculation of possible “iatrogenic” migration of the stone. Conventional hyperosmolar water-soluble contrast media have pharmacologic effects, including cholinesterase inhibition, the release of histamine, and in the gut, the release of a serotonin-like substance, which enhances bowel motility [2-4].

## Conclusions

We believe that the hyperosmolar contrast agent played a role in inducing osmotic pressure, intestinal smooth muscle contraction, and decreasing wall edema, which led to the spontaneous disimpaction and migration of the stone forwards. We believe this presentation is unique and warrants a heightened clinical awareness in order to avoid delayed diagnosis and management.

## References

[1] Reinhardt SW, Jin LX, Pitt SC, Earl TM, Chapman WC, Doyle MB (2013). Bouveret's syndrome complicated by classic gallstone ileus: progression of disease or iatrogenic?. J Gastrointest Surg.

[2] Cappell MS, Davis M (2006). Characterization of Bouveret's syndrome: a comprehensive review of 128 cases. Am J Gastroenterol.

[3] Ceresoli M, Coccolini F, Catena F, Montori G, Di Saverio S, Sartelli M, Ansaloni L (2007). Water-soluble contrast agent in adhesive small bowel obstruction: a systematic review and meta-analysis of diagnostic and therapeutic value. Br J Surg.

[4] Haddad FG, Mansour W, Deeb L (2018). Bouveret's syndrome: literature review. Cureus.

